# Self-motion sensitivity to visual yaw rotations in humans

**DOI:** 10.1007/s00221-014-4161-0

**Published:** 2014-12-16

**Authors:** Alessandro Nesti, Karl A. Beykirch, Paolo Pretto, Heinrich H. Bülthoff

**Affiliations:** 1 Department of Human Perception, Cognition and Action, Max Planck Institute for Biological Cybernetics, Spemannstraße 38, 72076 Tübingen, Germany; 2 AMST-Systemtechnik GmbH, Lamprechthausner-Str. 63, 5282 Ranshofen, Austria; 3 Department of Brain and Cognitive Engineering, Korea University, Seoul, 136-71 Korea

**Keywords:** Differential threshold, Vection, Self-motion perception, Yaw, Perceptual nonlinearities, Virtual reality, Psychophysics

## Abstract

While moving through the environment, humans use vision to discriminate different self-motion intensities and to control their actions (e.g. maintaining balance or controlling a vehicle). How the intensity of *visual* stimuli affects self-motion perception is an open, yet important, question. In this study, we investigate the human ability to discriminate perceived velocities of visually induced illusory self-motion (vection) around the vertical (yaw) axis. Stimuli, generated using a projection screen (70 × 90 deg field of view), consist of a natural virtual environment (360 deg panoramic colour picture of a forest) rotating at constant velocity. Participants control stimulus duration to allow for a complete vection illusion to occur in every single trial. In a two-interval forced-choice task, participants discriminate a reference motion from a comparison motion, adjusted after every presentation, by indicating which rotation feels stronger. Motion sensitivity is measured as the smallest perceivable change in stimulus intensity (differential threshold) for eight participants at five rotation velocities (5, 15, 30, 45 and 60 deg/s). Differential thresholds for circular vection increase with stimulus velocity, following a trend well described by a power law with an exponent of 0.64. The time necessary for complete vection to arise is slightly but significantly longer for the first stimulus presentation (average 11.56 s) than for the second (9.13 s) and does not depend on stimulus velocity. Results suggest that lower differential thresholds (higher sensitivity) are associated with smaller rotations, because they occur more frequently during everyday experience. Moreover, results also suggest that vection is facilitated by a recent exposure, possibly related to visual motion after-effect.

## Introduction

When moving through the environment, continuous variations of the retinal image (optic flow) often provide important self-motion cues and play a major role in self-motion perception (Von Helmholtz 1925; Gibson 1950). The importance of optic flow is particularly striking when conflicting information arises from the sensory systems involved in the perception of self-motion (mainly visual, vestibular and somatosensory systems). A frequently cited experience is the feeling of self-motion on a stationary train when a neighbouring train begins to move.

In everyday life, the intensity of self-motion varies over a wide range, from small subtle postural changes to stronger movements occurring during sport activities, for instance. Reliable estimates of these movements are obviously essential for a variety of crucial tasks (e.g. maintaining posture in the presence of external disturbances or controlling a vehicle). However, recent studies showed that the ability to estimate motion intensity varies with the intensity of motion stimuli (Zaichik et al. 1999; Mallery et al. 2010; Naseri and Grant 2012; Nesti et al. 2014). In these studies, differential thresholds (DTs), i.e. the smallest detectable changes in stimulus intensity, were measured over wide ranges of linear (e.g. 0–2 m/s^2^) and angular (e.g. 0–160 deg/s) motions. Results indicate that the relationship between DTs and stimulus intensity may be described by a power law, Δ*S* = k * S ^*b*^, where *S* is the stimulus intensity, Δ*S* is the DT, and *k* and *b* depend on the type of investigated motion (e.g. vertical translation or rotation). These works unequivocally show that DTs for self-motion in darkness are *not constant* but rather increase with motion intensity. In other words, motion sensitivity (i.e. the ability to detect small changes in stimulus intensity) worsens at higher motion intensities. This implies a *nonlinear* relationship between actual motion intensity and perceived motion intensity (Fechner 1860). Indeed, any sensory system whose sensitivity is *not constant* over the response range of the sensor is by definition *nonlinear*. This perceptual nonlinearity, shown for other sensory modalities (e.g. Teghtsoonian 1971), might reflect better sensitivity for ranges of stimulus intensities that are more frequent in everyday life (Stocker and Simoncelli 2006).

Despite the well-established role of visual cues in self-motion perception (Dichgans and Brandt 1973), less effort has been dedicated to measuring DTs for visual self-motion cues, perhaps because of the methodological challenge of ensuring that visual motion is indeed perceived as self-motion rather than object motion. DTs for visually simulated motion in depth supporting a Weber-like perceptual law were measured by Wei et al. (2010), to investigate how visual perceptual uncertainty affects balance responses. However, their choice for visual stimulation may indeed not fully address the issue of self-motion versus object motion for two reasons. First, the radial velocity of the random-dot flow field did not vary with eccentricity, as it would for vision during self-motion in a 3D environment. Second, the stimulus duration of 0.8 s is too short for a compelling visually evoked self-motion illusion (vection), which usually requires between 2 and 40 s, depending on the experimental conditions (see “Discussion”). In this study, we investigate human self-motion sensitivity by measuring DTs for visually evoked yaw rotation perception in an immersive virtual environment (circular vection). This constitutes a step forward in the understanding of self-motion sensitivity in more realistic conditions, where inertial and visual cues are both available. We hypothesize that DTs for vection increase with motion intensity following a trend described well by a power law, confirming the nonlinearity of self-motion perception observed when moving in darkness. This experiment will furthermore pave the way for a comparison between DTs for different combinations of visual and inertial cues and will lead to further investigation of the neural processes underlying self-motion perception and multisensory integration.

Studying the contribution of visual cues to self-motion perception requires great care in the design of the stimulus. Indeed, as previously mentioned, moving visual stimuli do not necessarily evoke a perception of self-motion. A well-established theory argues for a reciprocal relationship between the perception of object motion and self-motion (Dichgans and Brandt 1978), although other models have been suggested (Wertheim 1994). Moreover, several studies show that vection is modulated by the physical properties of the stimulus, as well as by cognitive factors. For example, the vection onset time (VOT) depends on the field of view of the visual stimulation and on the numbers of elements (e.g. dots) in the scene (Webb and Griffin 2003), while the sensation of vection may be enhanced by adding inertial vibration (Riecke et al. 2005) or by using a realistic, as opposed to unnatural, virtual environment (Riecke et al. 2005, 2006). In this study, a realistic visual stimulus rotating at constant velocity around the vertical axis of the participants was used. Note that constant velocity rotations are a particularly appropriate choice, as the insensitivity of the vestibular system to constant rotations strongly mitigates conflicting multisensory information. Moreover, participants also experienced stimulus-unrelated vibrations throughout the entire experiment (see “Methods” and “Discussion”).

Psychophysically measuring human self-motion DTs will allow improvements in the field of self-motion perception modelling, where the lack of experimental evidence has often led to the assumption of constant motion sensitivity (e.g. Bos and Bles 2002; Zupan et al. 2002; Newman et al. 2012). Improved model accuracy is beneficial for the design of simulation environments, such as motion simulators used for driver and pilot training. For instance, in vehicle simulation the motion of the simulated vehicles could be modified to better suite simulator capabilities as long as the manipulations remain unperceived (i.e. below DT). A better understanding of pilots’ perception over wide motion intensities also allows for more effective simulator training protocols in extreme conditions with the goal of better prediction and avoidance of accidents. Furthermore, in the medical diagnosis of balance disorders, psychophysical tests (Merfeld et al. 2010) could supplement currently used eye movement tests (Bárány 1921; Halmagyi and Curthoys 1988) in cases where eye movement cannot be measured (Merfeld et al. 2010) or to specifically measure perception since self-motion perception and ocular reflexes do not always match (MacGrath et al. 1995; Merfeld et al. 2005a, b; Wood et al. 2007).

## Methods

### Participants

Eight participants (aged 26–53 years, one female), five naïve and three authors (AN, KB and PP), took part in this study and gave their informed written consent prior to inclusion in the study, in accordance with the ethical standards specified by the 1964 Declaration of Helsinki. They all had normal or corrected-to-normal vision and reported no history of balance disorders and no susceptibility to motion sickness.

### Set-up

**Fig. 1:**
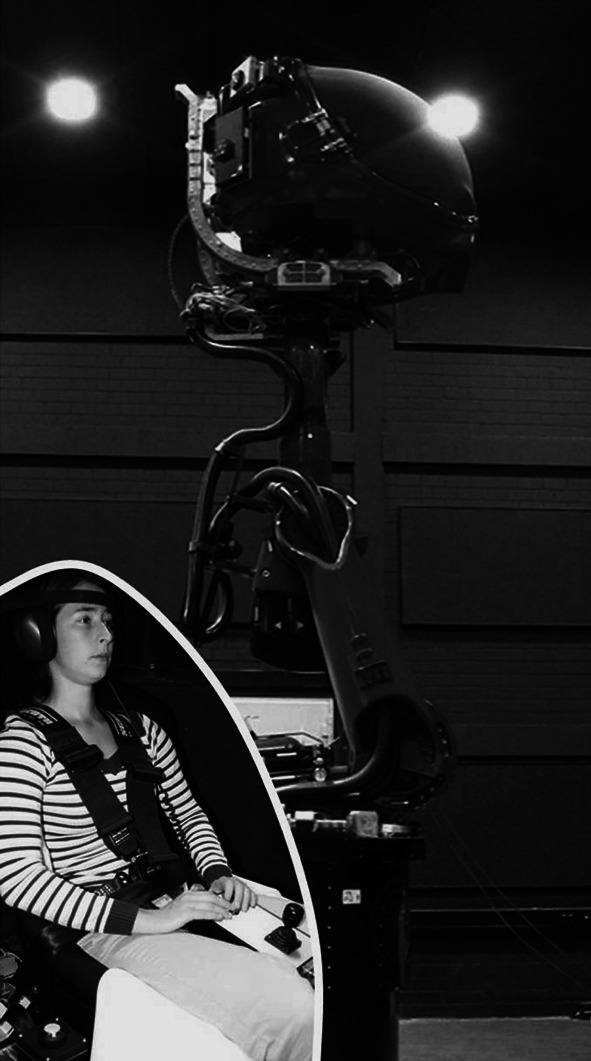
Experimental set-up. Participants sat inside the simulator’s cabin, where visual stimuli were presented on the inner surface of the cabin door by means of two projectors (stimulus resolution: 19.6 px/deg, refresh rate: 60 Hz, FOV: 70 × 90 deg)

The study was conducted using the Max Planck Institute CyberMotion Simulator (Fig. 1, for technical details refer to Nieuwenhuizen and Bülthoff (2013); Robocoaster, KUKA Roboter GmbH, Germany). Inside the closed cabin, two projectors (1,920 × 1,200 pixels resolution, 60 Hz frame rate) display on the white, curved inner surface of the cabin door, approximately 60 cm from the participants’ head. A field of view (FOV) of approximately 70 × 90 deg and an actual resolution of approximately 19.6 pixels/deg were used for the experiment. Participants were seated in a chair with a 5-point harness. They wore headphones playing white noise during the presentation of visual stimuli (Fig. 3) to mask external auditory cues. The head was restrained with a Velcro band, which combined with careful instruction to maintain an upright posture, helped participants to avoid coriolis effects (Guedry and Benson 1976; Lackner and Graybiel 1984), i.e. the sense of discomfort or nausea following head tilts during inertial rotations at constant velocity (see below). Bidirectional participant–experimenter communication was active throughout the experiment for safety. Participants interacted with the experiment using a button box with three active buttons, one to initiate and terminate the stimulus (control button) and the other two for providing a forced-choice response (response buttons, see “Procedure”). Eye movements were not recorded (see “Discussion”).

### Stimulus generation

**Fig. 2:**
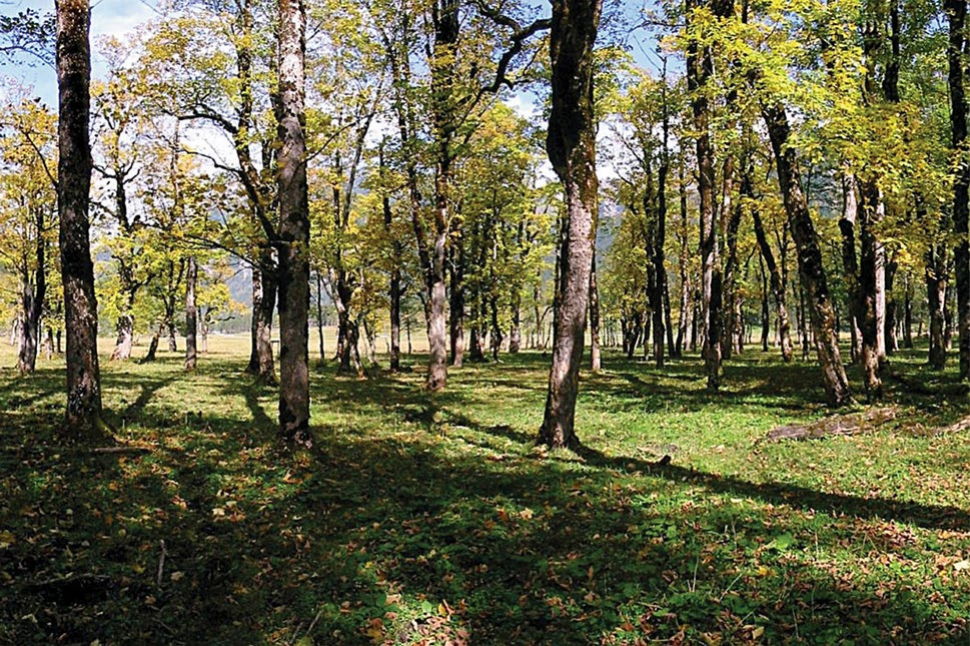
Fragment of the 360 deg panoramic colour picture used for generating the realistic virtual environment

**Fig. 3:**
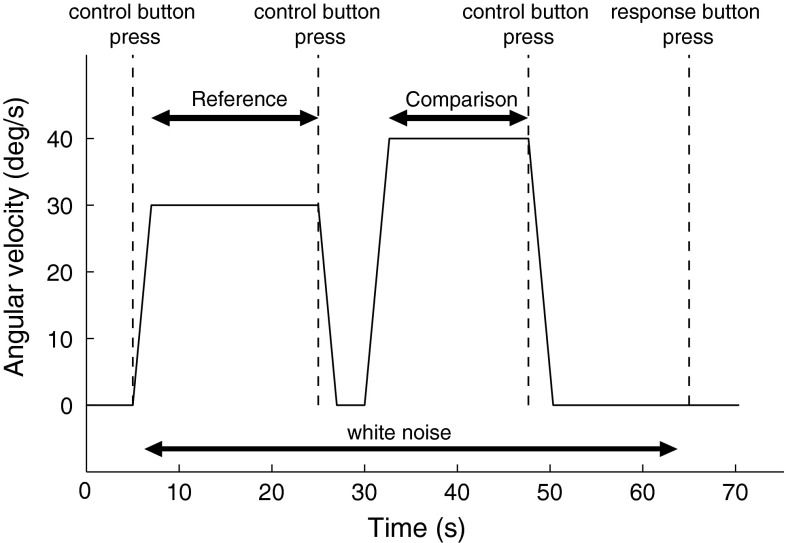
Angular velocity of the virtual stimulus during a typical trial. Note that the order was varied randomly (see text)

Visual stimuli were generated with authoring software for interactive 3D applications (Virtools, 3DVIA). A 360 deg panoramic picture of a forest (Fig. 2) was displayed on the surface of the cabin (60 cm away). In order to avoid motion parallax, the software projected the image on a cylinder created in the virtual environment whose axis coincides with the earth-vertical axis of the participants’ point of view in the virtual environment. The radius of the virtual cylinder (5 m) was chosen to achieve a satisfactory visual appearance on the screen (i.e. texture resolution and object size). To preserve the participants’ natural behaviour, no visual fixation was used. Stimuli consisted of rotations of the virtual cylinder around its axis with constant rotational velocities in the range of 5–72 deg/s. A constant linear acceleration onset/offset was generated, lasting 2 s for the reference stimulus and slightly longer for the comparison due to its higher velocity (see Fig. 3). The onset/offset ramp resulted in a more tolerable and natural motion sensation, as compared to a step onset/offset.

During each session, participants were continuously rotating around the head-centred vertical axis at the constant velocity of 20 deg/s so as to generate vibrations unrelated to the stimulus. Note that the perception of constant inertial rotations disappears within a few seconds after rotation onset (Bertolini et al. 2011), and even the small otoliths stimulation is sub-threshold.^1^ However, the vibrations resulting from the simulator’s motion have been shown to enhance VOT and convincingness (Riecke et al. 2005). The absence of centripetal accelerations was monitored with a 3-axis accelerometer placed on the top of a participant’s head. Rotation direction was reversed approximately every 15 min corresponding to session breaks (see below), and stimulus presentation began 1 min after constant velocity was reached, allowing for the sensation of rotational motion to disappear. To avoid confusion, throughout the paper we refer to the visual stimuli in a frame of reference relative to the rotating participants.

### Procedure

Each trial was composed of two consecutive presentations of the visual stimulus, separated by a pause of 3 s (see Fig. 3). The constant velocity amplitude of one of the presentations (reference stimulus) remained the same across all trials, while the amplitude of the comparison was systematically varied. Reference and comparison had opposite directions, as this was found to hinder comparison of purely visual (object) velocities in pilot work. Such a comparison might in fact artificially lower the thresholds, facilitating the discrimination task without, however, contributing to self-motion perception. Presentation order was randomized to prevent complications due to order effects and visual motion after-effects (Hershenson 1989).

Prior to each trial, the virtual environment was visible and stationary in front of the participants. They initiated each trial by pressing the control button. The visual environment was then rotated at constant velocity. Participants were in control of the stimulus duration and were instructed to terminate it by pressing the control button when they confidently perceived the virtual scene as stationary. According to the “reciprocity” theory between object and self-motion (Dichgans and Brandt 1978), this is equivalent to confidently perceiving themselves being rotated within a *stationary* scene, with all the visual motion attributed to self-motion. After both stimuli of the trial were terminated, the screen turned black and participants were asked to report “which rotation was faster” by pressing one of the two response buttons (first or second). No feedback on the correctness of the response was provided. The time to scene stationarity (TSS), here defined as the time between stimulus onset and stimulus termination, was recorded for each stimulus with a resolution of 1 ms. After each trial, participants were allowed to rest, the virtual environment remained visible and stationary in front of them, and no white noise was presented.

**Fig. 4:**
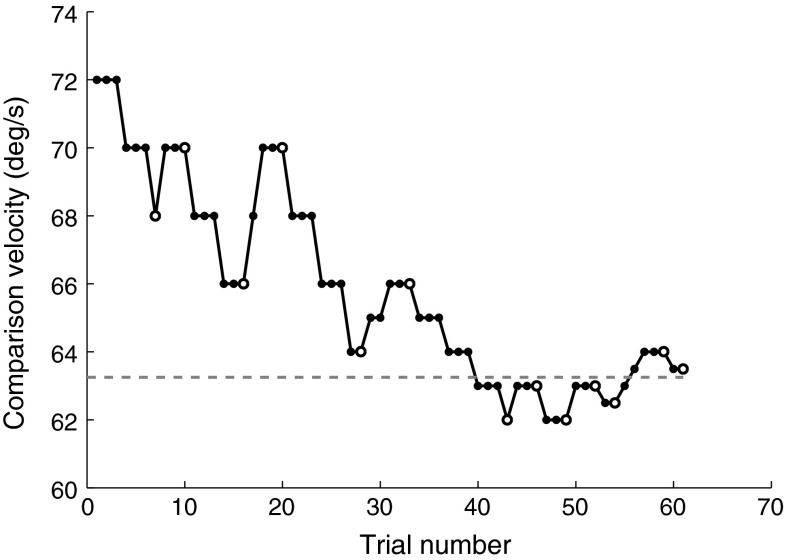
Comparison velocities for each trial of a typical experimental session with a reference velocity of 60 deg/s. Empty circles indicate reversal points. The last eight reversals were averaged to compute the DT (*dashed line*)

The experiment was divided into five sessions of approximately 45 min each, with breaks of approximately 5 min every 15 min of experiment to avoid fatigue. Participants completed the experiment over five different days (1 condition per session per day, order randomized). In each condition, the participants’ DT was measured for a different reference velocity (5, 15, 30, 45 and 60 deg/s) using a psychophysical two-interval forced-choice (2IFC) procedure. Comparison velocities were adjusted for every trial according to an adaptive staircase algorithm which decreased the stimulus level after three consecutive correct responses and increased it after every incorrect response [3-down–1-up rule (Levitt 1971)]. This algorithm eventually converges to the stimulus level where a stimulus increase (wrong answer) or decrease (3 consecutive correct answers) is equally probable (*p* = 0.5), meaning that the probability of a single correct answer is 0.794 (cubic root of 0.5). For the 5 deg/s reference condition, the initial comparison velocity was 7 deg/s, with a step size of 0.2 deg/s, halved after the staircase reversed direction five times. For the other conditions, the initial comparison velocity *c*_0_ was set according to the formula *c*_0_ = 6/5 * ref_v, where *ref_v* is the reference velocity. The step size, initially set at 2 deg/s, was halved after five reversals (1 deg/s) and again after 10 (0.5 deg/s). Every session was terminated after 13 reversals. The comparison velocities over one experimental session for a typical participant are illustrated in Fig. 4.

### Data analysis

DTs were obtained from each condition by averaging the last eight staircase reversals (Fig. 4). An alternative estimate of DTs was obtained from the least-squares estimation (LSE) of a psychometric function to the participant’s responses for every condition. In this case, DTs were defined as the reference velocity increment necessary for a 0.794 probability of correct discrimination. This allowed for investigating, using an ANOVA, whether DT estimates could be affected by the formula chosen for their calculation. Linear regression analysis was performed on DTs to test whether human vection sensitivity depends on motion intensities. A repeated measures ANOVA was used to test for differences in the TSSs between the two levels of the factor “presentation order” (first or second) and between the five levels of the factor “stimulus intensity”, corresponding to the five different reference velocities. Statistical analyses were performed in MATLAB (2012a) using the statistical toolbox. Effects are considered statistically significant if their *p* value is <0.05.

Two different models, a Weber’s law (Fechner 1860) and a power law (Guilford 1932), proposed in the literature to relate DTs to stimulus intensity, were fit to the data. The Weber’s law function has the general form Δ*S* = *k* * *(S* + *a)*, where Δ*S* is the DT, *S* the stimulus intensity, *k* the Weber fraction and *a* represents the amount of noise that exists when the stimulus is zero (Gescheider 1997), while the power law function has the form Δ*S* = k * S ^*b*^. The two models describing rotational vection sensitivity were compared based on their goodness of fit, measured by the coefficient of determination *r* ^2^.

## Results

Each condition took approximately 45 min and required on average 63 trials. No session needed to be terminated because of fatigue or other reasons, although mild symptoms of motion sickness were often reported (see “Discussion”).

**Fig. 5:**
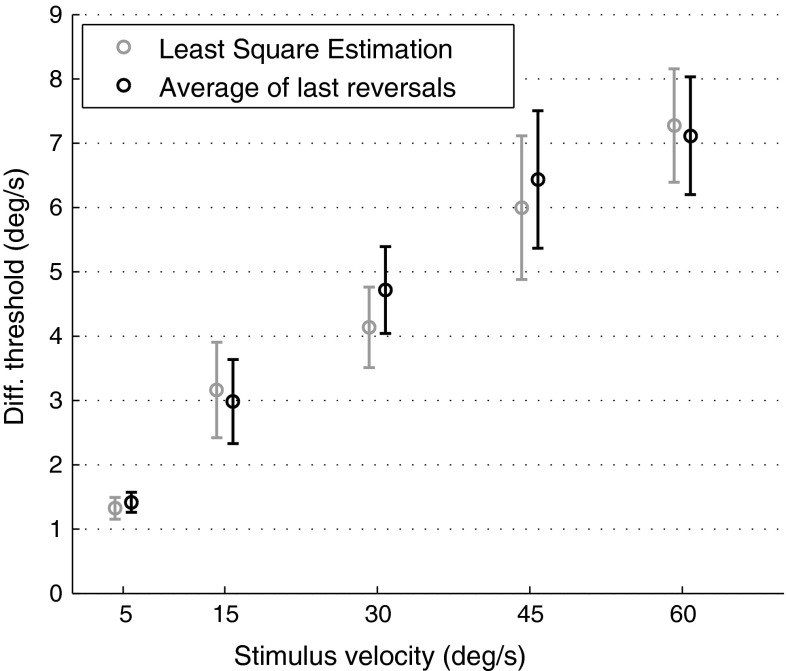
Comparison between DTs estimated by LSE and by the average of the last eight reversals. *Error bars* represent standard errors of the mean

As illustrated in Fig. 5, no difference is found between DTs obtained by reversal averaging and by LSE [*F*(1,7) = 2.62, *p* = 0.15]. We therefore proceeded to analyse the former estimates, as they do not require an assumption on the shape of the psychometric function. Indeed, such an assumption cannot be properly done because the adaptive procedure concentrates stimulus presentations only around its region of convergence (0.79 probability of correct discrimination, see “Methods”).

**Fig. 6:**
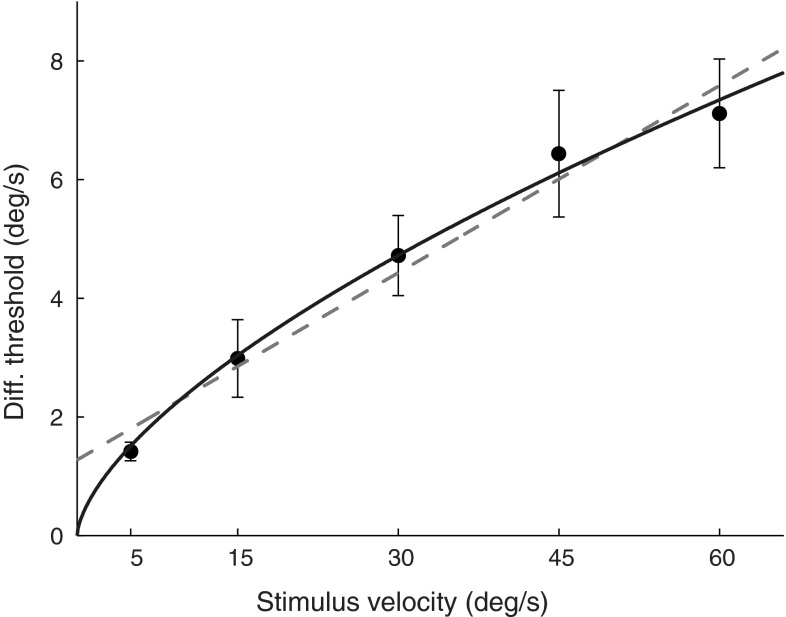
DTs for head-centred yaw rotations increase with stimulus intensity following a trend well described by a concave power law (*continuous line*). A Weber’s Law function (*dashed line*) provides a slightly poorer fit. *Error bars* represent standard errors of the mean

DTs for vection are presented in Fig. 6. Regression analysis yielded a slope coefficient of 0.11 ± 0.017, indicating that DTs increase with motion intensity [*t*(38) = 6.30, *p* < 0.001].

Fitting the data with a Weber’s law function in the form Δ*S* = k * (S + *a)* resulted in the coefficients *k* = 0.11 and *a* = 12.12, whereas from the fit of a power law function (Δ*S* = k * S ^*b*^), the coefficients *k* = 0.54 and *b* = 0.64 were obtained. Coefficients of determination are *r* ^2^ = 0.97 and *r* ^2^ = 0.99 for the Weber’s and power law, respectively, indicating that the both functions provide a good description of the measured DTs.

**Fig. 7:**
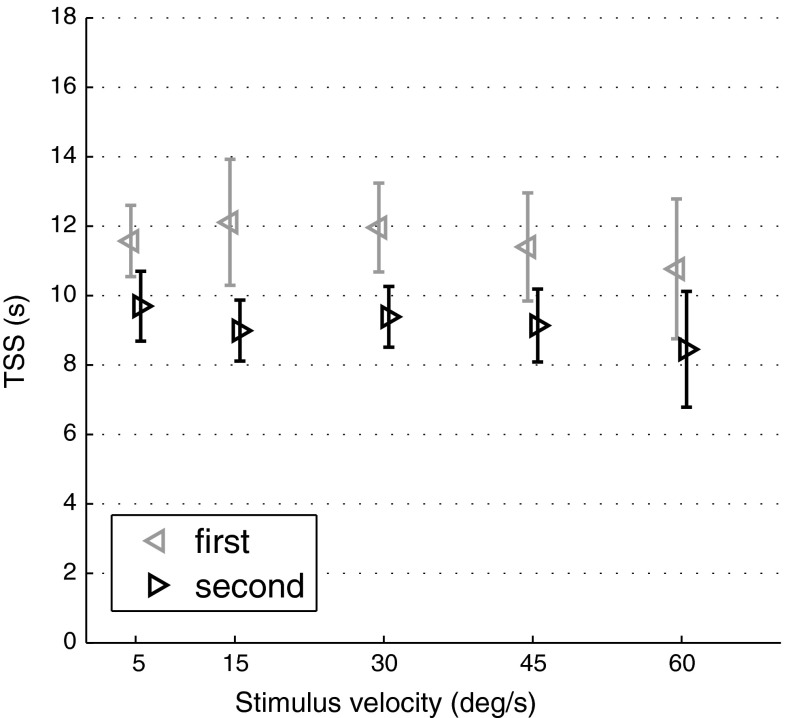
TSSs are on average independent from the visual motion intensity. Participants took a statistically significantly longer time for terminating the first rather than the second stimulus. *Error bars* represent the standard errors of the mean

Average TSSs (Fig. 7) do not significantly depend on the motion intensity [*F*(4, 28) = 0.16, *p* = 0.96]; however, they significantly depend on presentation order [*F*(1, 7) = 10.43, *p* = 0.015], with shorter TSSs for the second stimulus of each trial (see Fig. 7). Average TSSs across all conditions are 11.56 and 9.13 s for the first and second stimulus presented, respectively.

## Discussion

In this study, we investigated human sensitivity to visually induced self-motion perception as evoked by immersive visual stimulation. We found that DTs increase with stimulus intensity (i.e. rotational velocities), indicating that sensitivity to circular vection is not constant over the investigated motion intensity range, but rather worsen at greater velocities. This represents a nonlinearity in the perception of self-motion. Such perceptual nonlinearity also emerges from psychophysical studies on linear vection discrimination (Wei et al. 2010) and magnitude estimation (Brandt et al. 1973; Dichgans and Brandt 1973). Perceptual nonlinearities may also explain why postural responses evoked by visual stimulation do not continuously increase with stimulus amplitude but rather saturate (Wei et al. 2010; Van der Kooij et al. 2001). As suggested by Wei et al. (2010), this could reflect a Bayesian integration by the CNS of visual and inertial cues, assigning less weight to stronger as compared to weaker visual motions as they have greater uncertainty (i.e. higher DTs). Interestingly, nonlinear behaviour in response to rotational optic flow is not shown in electrophysiological recordings from neurons in MSTd (Tanaka and Saito 1989) and in the vestibular nuclei (Dichgans et al. 1973; Henn et al. 1974; Waespe and Henn 1977), where average firing rates linearly depend on stimulus velocity. Similarly, vestibular and optokinetic reflexes, responsible for stabilizing the gaze in response to head rotations, show an approximately linear response over the investigated range of visual (Paige 1994) and inertial (Pulaski et al. 1981; Weber et al. 2008) rotational velocities. A possible reconciliation between physiological linearity and perceptual nonlinearity might relate to the increased firing rate variability observed in the vestibular nuclei for stronger compared to weaker self-motion intensities (Dichgans et al. 1973; Henn et al. 1974; Allum et al. 1976; Waespe and Henn 1977). Indeed, higher variability of the sensory signals leads to less precise perceptual discriminations (higher DTs) even when the average sensory response (firing rates) over multiple repetitions remains linear. Further investigation is, however, required in order to relate neurophysiological responses to psychophysical thresholds for self-motion.

Both the power law model and the Weber’s law model provided an excellent fit for average DTs over the investigated motion range. Nevertheless, it is known that for a large range of sensory input amplitudes, Weber’s law does not hold (Teghtsoonian 1971) and the power law captures the changes in DTs better (Guilford 1932). This suggests that differences in model accuracy will arise when measuring DTs for stronger and/or weaker yaw rotation intensities, with the power law model becoming the preferable alternative. We did not measure DTs for reference velocities higher than 72 deg/s (corresponding to 1.2 deg/frame in our set-up) because, as confirmed by preliminary testing on two different participants, visual rotations faster than 1.2 deg/frame resulted in a visual blurriness that prevents vection from arising in our set-up. Furthermore, we noticed that rotations slower than 5 deg/s did not succeed in evoking full vection within reasonable stimulus exposure times (approximately 60 s). Note, however, that both models allow one to safely conclude that DTs significantly increase with stimulus intensity and thus that the perception of self-motion from visual cues is nonlinear.

Self-motion perception arises from central processing of inertial (vestibular and somatosensory) and visual information. This also includes dissociation of the visual features related to self-motion (optic flow) from those related to other events (e.g. object motion). Therefore, in a virtual environment, a coherent perception of self-motion is only possible through a careful design of the structural and temporal properties of the visual stimulus, where the design should also consider their interaction with stimuli of other sensory modalities. In this study, particular care was taken to address this point, resulting in the following choices. The use of a forest rather than vertical bars (Brandt et al. 1973) or a dot field (Berthoz et al. 1975) was motivated by studies showing that a natural stimulus decreases VOT and TSS, and increases immersion in a vection task (Riecke et al. 2005, 2006). A ramp onset of the visual motion was chosen over a step onset because, according to outlier detection models of perception (Wei and Körding 2009), sudden changes of the visual environment (step onset) are more likely to be due to movements of the surrounding and therefore neglected for estimating self-motion, whereas gradual changes in self-motion velocities are a more natural self-motion stimulus. Subjective reports of two participants experiencing both types of onset additionally confirmed that a ramp onset generates a more tolerable multisensory conflict. We employed constant velocity visual rotations around the vertical axis of the participants, a stimulus that minimizes sensory conflicts (see below) and therefore favours a coherent self-motion perception. The lack of visual fixation, combined with careful instruction to look ahead, favoured immersion in the virtual environment, while at the same time allowed for peripheral stimulation and optokinetic nystagmus, thereby avoiding complications due to the Aubert-Fleischl paradox (De Graaf et al. 1991). Given the considerable individual differences in VOTs (Brandt et al. 1973; Berthoz et al. 1975; Riecke et al. 2005, 2006), the duration of stimulus presentations was self-paced, i.e. participants were instructed to terminate the stimulus only after perceiving the visual scene as stationary (complete self-motion illusion). Finally, stimulus offset also followed a ramp profile, with the visual stimulus slowing down to a stationary visual pattern. This, again, resembles a more natural self-motion pattern than a sudden stop and mitigates both nausea and visual motion after-effect. Overall, we believe that this paradigm is well-suited for evoking vection and sets a useful precedent for self-motion studies in visual environments.

Average TSSs were 11.56 and 9.13 s for the first and second stimulus presented, respectively. These values are consistent with Brandt et al. (1973), where a pure sense of self-rotation was perceived on average 8–12 s after stimulus onset. Note, however, that average VOTs in the literature show high variability, ranging between 2 and 40 s depending on stimulus properties and cognitive factors such as visual FOV, vibrational cues and scene naturalism (Brandt et al. 1973; Berthoz et al. 1975; Webb and Griffin 2003; Riecke et al. 2005, 2006). Similar dependences are therefore to be expected also in TSSs. In agreement with VOTs reported by Brandt et al. (1973) and Berthoz et al. (1975), we found no significant dependency of TSSs on stimulus intensity. This result differs, however, from the result found by Riecke et al. (2006). We additionally report a significant effect of presentation order on TSSs, suggesting that vection arises more easily shortly after previous exposure. A similar observation is reported in Berthoz et al. (1975). The visual motion after-effect is a reasonable explanation for this result: any potential residual motion perception following the first stimulus is expected to shorten the TSSs. In this study, the inter-trial pause of 3 s was chosen as a compromise between keeping a vivid impression of the 1st stimulus and the 4 s time constant of the visual motion after-effect reported by Hershenson (1989) for a 20-s constant rotation stimulus. It is therefore possible that for some trials the residual self-motion perception after the first stimulus had not yet decayed to 0 deg/s when the second stimulus began. Note, however, that this has no repercussion on DT estimates as it does not affect the perceived self-motion intensity at the moment of the button press (i.e. when the illusion is complete and all the visual motion is attributed to self-motion).

During the course of each experimental session, all participants reported mild symptoms of motion sickness. They consistently reported the discomfort being provoked by the onset/offset of the visual stimuli, whereas the constant velocity of the visual rotations was better tolerated. This fact is explained by vestibular and somatosensory dynamics (which respond to inertial accelerations) and the sensory conflict hypothesis (Beadnell 1924) underlying the most widely accepted motion sickness theories and models (Reason 1969; Oman 1982). According to this theory, motion sickness arises whenever visual and vestibular sensory cues deviate from normal daily patterns and conflict with each other. Such a conflict was minimized to a great degree in our study by employing visual and inertial rotations at constant velocity, where a lack of angular and linear inertial accelerations (other than gravity) allows for non-conflicting sensory information from the visual and inertial sensory systems. However, the onset/offset of the visual stimulus presented a visual acceleration not matched by any physical acceleration, thus resulting in sensory information interpreted as conflicting by the central nervous system.

In this study, we chose to measure DTs for stimuli rotating at constant velocity (0 Hz) as higher frequencies of the visual stimulus would likely generate a sensory conflict which prevents participants from perceiving self-motion. However, constant rotations do not frequently occur in everyday life, and it is therefore legitimate to question the generalizability of the results. Constant visual rotations elicit sustained neural responses in the vestibular nuclei of alert monkeys, whereas responses to transient visual velocities are attenuated (Waespe and Henn 1977). This behaviour is often referred to as low-pass behaviour because only the lower frequencies of the input signal are maintained in the response. As demonstrated by Robinson (1977) using a modelling approach, neural responses in the vestibular nuclei to visual-only and inertial-only rotations (Waespe and Henn 1977) add linearly, with responses to rotations in darkness showing a high-pass behaviour complementing the low-pass behaviour of the visual responses. Consequently, rotations in the light show both transient and sustained activity. This would indicate that vision mainly contributes to the perception of self-motion at low frequencies [beginning to attenuate with increasing frequencies at about 0.03 Hz (Robinson 1977)], where the response of the inertial systems (e.g. the semicircular canals of the vestibular system] is either strongly attenuated or absent (Robinson 1977). Results from the present work are therefore expected to generalize well to transient profiles in a range of frequencies present in natural movements (Grossman et al. 1988). It should be noted that visual stimuli might contain additional information that, although not directly related to the perception of self-motion, could help in discriminating different motion intensities. For instance, short or periodic movements allow to judge the intensity of the motion based on the travelled distance of specific image features (e.g. a tree), a visual task that is not informative about the self-motion experienced by the participants, who could be able to perform such task even without perceiving any self-motion. We expect that, in the presence of such cues, DTs will be artificially lower; this does, however, not detract from the main conclusion that self-motion perception nonlinearly depends on motion intensity.

Nonlinearities in the perception of inertial, rather than visual, yaw rotation stimuli have also been investigated (Mallery et al. 2010). The measured DTs, defined as we did here at 79 % chances of correct discrimination, are only slightly smaller (approximately 2, 3 and 5 deg/s for reference stimuli of 20, 40 and 60 deg/s, respectively) and are similarly described with a convex power law. These similarities suggest a common neural mechanism acting on the internal representation of self-motion within the central nervous system. Future studies need to systematically compare self-motion sensitivity for inertial-only, visual-only and congruent visual–inertial cues. Beside the high ecological validity of these studies (natural movements often provide multisensory cues over wider intensity ranges), such comparison will inform the type and location of the neural processes underlying self-motion perception.

## Footnotes

1 Assuming that the otolithic organs are located at 10 cm from the centre of rotation, the centripetal acceleration sensed by the otoliths is: *F* = *r**(angular velocity)^2^ = 0.1 m*(20 deg/s*pi/180 deg)^2^ = 0.012 m/s^2^. This constant acceleration is below perceptual thresholds see Nesti et al. (2014) for review.
